# Epidemiology of Sports Related Concussion in Brazilian Jiu-Jitsu: A Cross-Sectional Study

**DOI:** 10.3390/sports7020053

**Published:** 2019-02-25

**Authors:** Matthew Spano, Donald A Risucci, Mill Etienne, Kristina H. Petersen

**Affiliations:** 1 Department of Undergraduate Medical Education, New York Medical College, Valhalla, New York, NY 10595, USA; 2 Department of Undergraduate Medical Education & Department of Surgery, New York Medical College, Valhalla, New York, NY 10595, USA; Donald_Risucci@nymc.edu; 3 Department of Neurology, New York Medical College, Valhalla, New York, NY 10595, USA; Mill_Etienne@nymc.edu; 4 Department of Biochemistry & Molecular Biology, New York Medical College, Valhalla, New York, NY 10595, USA; K_HarrisPetersen@nymc.edu

**Keywords:** Brazilian Jiu-Jitsu, BJJ, concussion, sport-related concussion, martial arts

## Abstract

Brazilian Jiu-Jitsu (BJJ) is a rapidly growing grappling sport with a wide spectrum of participants. This cross-sectional study examined the lifetime prevalence of concussion in adult BJJ practitioners in the United States using a 17-item survey. A total of 778 (11.4% female) BJJ practitioners with a median age of 31 years completed the survey. Overall, the lifetime prevalence of the self-reported BJJ-related concussion was 25.2%. However, the prevalence was higher among females than males (43.0% versus 22.9%; X^2^(1,740) = 15.129; *p* < 0.001). Factors independently associated with significantly increased odds of having sustained a BJJ-related concussion included a prior history of concussion (OR 1.76, 95% CI 1.14–2.74; *p* = 0.011) and female gender (OR 1.95, 95% CI 1.04–3.65; *p* = 0.037). The median return to sports time was three days, with 30.3% of participants returning on the same day as being concussed. The present study represents the first epidemiological research examining the concussions in BJJ. The results underscore the need for increased education on concussions and return to sports guidelines among BJJ coaches and practitioners.

## 1. Introduction

Brazilian Jiu-Jitsu (BJJ) is a rapidly growing grappling sport with a wide spectrum of participants. In contrast to combat sports, BJJ focuses on submissions such as joint locks, chokeholds, and control rather than punches and kicks. The goal is to control and submit the opponent as opposed to striking and inducing trauma. This lack of purposeful trauma distinguishes BJJ from combat sports such as boxing and mixed martial arts. 

Like other contact grappling sports, there is a risk for concussion. According to the most recent international consensus statement on concussion in sports, a sports-related concussion was defined as “a traumatic brain injury induced by biomechanical forces” [1]. Sports-related concussion is typically a brief self-resolving impairment in neurologic function. The derangement tends to be functional rather than structural in nature, and therefore is not often seen with neuroimaging studies. The symptoms of a sports-related concussion are broad and varied and do not have to include loss of consciousness (LOC) [1]. Return to sports and time to recovery from symptoms are also commonly studied among concussed athletes. Athletes with concussions who report more than four symptoms are at an increased risk for symptoms lasting greater than one week [2]. 

There has been limited research of injuries acquired from participation in BJJ. Within the current body of published research, the focus has been on orthopedic injuries and skin infections [3,4,5]. It has been observed that these injuries occur more often during practice than competitions [5]. Previous research has shown the average BJJ practitioner competes in 2.18 competitions per year and trains 7.63 h per week [5]. The paucity of competitions relative to practice time is likely a factor in the majority of injuries occurring during practice. The current body of published literature does not include studies that examine the prevalence of concussions among BJJ participants. Exploring the epidemiology of concussion in BJJ may help determine the best strategies for identification, treatment, and prevention of concussions within this sport.

Epidemiological studies on the period prevalence of concussions have been carried out in similar grappling sports such as wrestling. In the National Collegiate Athletic Association (NCAA) wrestling from 2005 to 2006, concussions represented 5.8% of all injuries or 0.42 concussions per 1000 athlete exposures [6]. During 2011–2015, the concussion rate in NCAA wrestling was reported to be 0.89 per 1000 athlete-exposures [7]. Data from the NCAA Injury Surveillance System reveal a steady increase in concussion rates across all sports, effectively doubling from 1988 to 2004 [8]. This increase is most likely to be due to the increasing awareness and reporting; it is, therefore, difficult to compare concussion prevalence estimates over this time period.

A recent study using retrospective self-reporting found the lifetime prevalence of concussion in water polo to be 36% [9]. The NCAA has carried out similar retrospective self-reporting studies. Among men’s contact sports, the concussion prevalence were 17.8% and 19.5% in lacrosse and wrestling, respectively [10]. Among women’s contact sports, which did not include grappling sports, the highest concussion prevalence was in ice hockey at 20.9% [10]. This data set was limited to concussions incurred during an athlete’s NCAA career. 

The vast majority of sport-related concussion research focuses on athletes under 30 years of age [8,10,11,12]. With a significant population of BJJ participants older than 30, there is an opportunity to analyze concussions in a population that are studied less frequently [4]. It has been shown that musculoskeletal injuries in BJJ occur more frequently in practitioners over age 30, although there has been no research published on brain injuries in this age group [13]. Outside of BJJ, there is evidence that females are more likely to suffer concussions in a given sport than their male counterparts [12]. To our knowledge, this phenomenon has yet to be studied within BJJ. 

The present study aimed to quantify the lifetime prevalence of concussions among BJJ practitioners to identify factors associated with greater odds of having a concussion. We hypothesize that the lifetime prevalence of concussions will be similar to other grappling sports. We also expect the practitioners who participate in competitions and those who are older to have a greater prevalence of concussions and take longer to return to sports. 

## 2. Materials and Methods

### 2.1. Study Population and Sampling

A web link to the survey was directly emailed to 42 BJJ coaches or school owners and directly posted on the social media websites of approximately 75 BJJ clubs and state groups located in the United States. Email contacts for school owners based in the United States were collected alphabetically by club name from the International Brazilian Jiu-Jitsu federation website [14]. The social media distribution focused on state-based BJJ Facebook groups. For example, the survey was posted publicly to the “Utah Jiu-Jitsu” Facebook page and members were invited to respond. This was repeated for each state’s BJJ page, with the majority of states having such a forum. Some states had more than one page, and the survey was posted on all identified pages in such instances. Any practitioner over the age of 18 was invited to complete the survey. The number of people receiving the invitation to complete the survey was unknown and the response rate is, therefore, indeterminable. The survey was disseminated via a link to the SurveyMonkey collection platform, set to prevent multiple responses from the same IP address to avoid repeat responses. 791 practitioners sent back completed or partially completed surveys; 13 surveys were excluded because the practitioners identified themselves as under the age of 18.

### 2.2. Survey

The survey consisted of 17 questions, beginning with demographic data and previous concussion information. Three participants known to the author completed the questionnaire and provided feedback on any ambiguity; changes were made for clarification prior to distribution. Responses from these three participants to the revised survey were included in the data set. Reliability and Validity of the survey were not formally tested. We asked for the respondents’ age, gender, number of previous concussions, and number of times being “knocked out or unconscious from a concussion.” The next section included questions about the respondents’ involvement in BJJ: length of time spent training, the number of practices attended per week, and the total number of lifetime competitions. 

The third section of the survey asked whether each practitioner had experienced one or more concussions as a result of participating in BJJ. Within the survey, participants were given a broad definition of a concussion as “a blow to the head or body followed by a variety of symptoms that may include any of the following: a headache, dizziness, loss of balance, blurred vision, ‘seeing stars,’ feeling in a fog or slowed down, memory problems, poor concentration, irritability, emotional outbursts, slurred speech, disturbed sleep pattern, nausea, or throwing up.” The survey explicitly stated that getting “knocked-out” or being unconscious does not always occur with a concussion. If the respondent reported having experienced a concussion, they were asked to identify which of the previously listed symptoms they had experienced. They were then asked how long after the concussion they returned to BJJ practices, how long they were asymptomatic before returning to BJJ, and whether they experienced a second BJJ-associated concussion at a later date. The full survey is available in the Supplementary Materials.

### 2.3. Data Processing

As the survey had both free-text and nominal fields, some data processing had to be done to consistently define participants’ answers. Similar data processing has been performed in other studies using retrospective surveys [9].

If the participant listed a number as “20+” it was counted as 20. If a range of numbers was given, the median between the two numbers was used. If the participant stated “about 3 years” it was counted as 3 years. A response of “a few days” was counted as 4 days. However, if the participant stated “too many to count” or “many” they were considered non-responders. Since not every participant responded to every question, percentages were calculated based on the number of participants who responded to a given question. 

### 2.4. Analysis

Statistical analysis was performed using the IBM developed Statistical Package for the Social Sciences (SPSS) (Armonk, NY, USA). Potential factors associated with report/non-report of a concussion were tested using separate Chi-Square (X^2^) tests for categorical variables and Student t-tests for continuous variables. Variables with associated p-values of less than or equal to 0.10 were included in multiple logistic regression analysis to identify those independently associated with a reported concussion. Variables were entered simultaneously into the regression model. Multicollinearity of variables in the equation was evaluated by examining Variance Inflation Factors (VIF) derived from sequential multiple linear regression analysis in which each variable was regressed on all others. A p-value of less than 0.05 was the marker for significance. Competition density was calculated for each respondent by dividing the number of reported lifetime BJJ competitions by the number of months training. Descriptive statistics will include reporting the median with interquartile range (IQR) for non-normally distributed variables.

## 3. Results

### 3.1. Demographics

Demographic information was collected from the 778 survey respondents. Females represented 11.4% of total respondents. Respondents had a median age of 31 (IQR: 13; Range 18–67) and reported practicing BJJ a median of 3 (IQR: 1.5; Range 1–7) days per week (Table 1). The median number of lifetime competitions per respondent was 2 (IQR: 6; Range: 0–100).

### 3.2. Concussions before BJJ

Before starting BJJ, 43.3% of respondents reported having at least one concussion. The lifetime prevalence of concussion was determined within both gender and age categories. While 43.7% of males reported a concussion prior to participating in BJJ, 39.3% of females reported a concussion prior to participating in BJJ (X^2^ (2, n = 774) = 1.930, *p* = 0.381). Concussion prevalence before starting BJJ increased significantly as age increased (X^2^ (5, n = 774) = 26.747, *p* < 0.001). Of those who reported having a concussion prior to participating in BJJ, 52.2% reported having at least one episode of LOC from head trauma. 

### 3.3. Concussions While Engaged in BJJ

A total of 187 (25.2%) practitioners reported a concussion while engaging in BJJ. Females reported significantly more concussions than their male counterparts (43% versus 22.9%; X^2^ (2, n = 187) = 19.222, *p* < 0.001). There were no differences in concussion prevalence across age groups (X^2^ (5, n = 187) = 6.044, *p* = 0.302). Logistic Regression Analysis (Table 2) indicated that female gender, reporting at least one concussion prior to starting BJJ, total number of competitions, and competition density was independently associated with greater odds of suffering a concussion when participating in the sport. VIF for variables in the equation ranged from 1.009 to 1.21 suggesting an acceptable level of multicollinearity.

### 3.4. Concussion Symptoms

**Figure 1:**
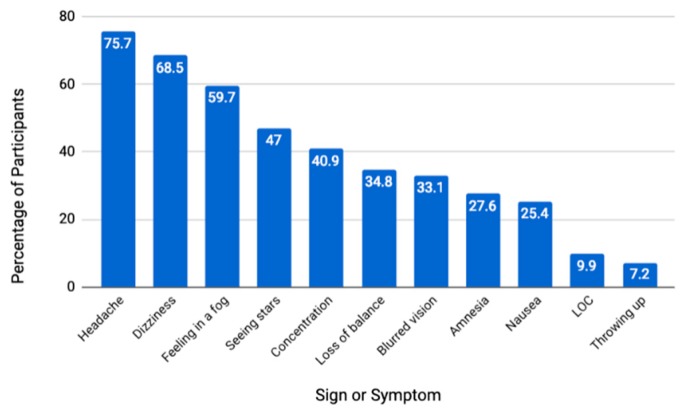
The Proportion of Respondents Experiencing BJJ-Related Concussion (n = 187) Reporting Specific Signs or Symptoms. LOC: Loss of Consciousness.

**Figure 2:**
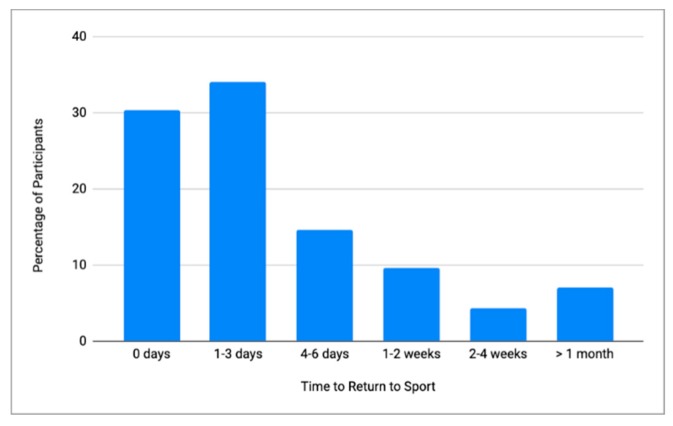
Time to Return to Sports after First Concussion.

Among the participants who reported experiencing a concussion while engaged in BJJ, we analyzed the symptoms they reported qualitatively. Among participants who reported experiencing a BJJ-related concussion, the median number of signs and symptoms was 4 (IQR: 4; Range: 1–11). The most commonly reported signs or symptoms were a headache (75.7%) and dizziness (68.5%) (Figure 1). The median return to sports time irrespective of symptoms was three days, with 30.3% of participants returning on the same day as their reported concussion (Figure 2). Among practitioners who reported a concussion while engaging in BJJ, 26.7% reported a second concussion from BJJ.

## 4. Discussion

As a grappling sport, BJJ puts practitioners at risk for incurring a sports-related concussion. In this retrospective survey, 25.2% of BJJ practitioners report having a concussion while engaging in BJJ in their lifetime. To our knowledge, this is the first study that attempts to record the prevalence of sports-related concussions in BJJ. Several findings were consistent with the recent studies in other sports. For example, the concussion prevalence of 25.2% in this study was similar to the career prevalence of 19.5% reported in NCAA men’s wrestling [10]. The reported prevalence in NCAA men’s wrestling covers the athletes’ collegiate career, typically ranging from one to five years. It may be more accurate to compare the lifetime prevalence in BJJ to lifetime prevalence in other sports. In a recent study of water polo athletes, 36% of participants reported at least one lifetime concussion in retrospective surveys [9]. When compared to older national studies, the increase in concussion prevalence found in this study is likely secondary to a more inclusive concussion definition, increased concussion awareness, and the methodology of retrospective surveys. In an anonymous survey, 22% of college athletes indicated that they would be unlikely to report concussion symptoms to a coach or athletic trainer [15]. Furthermore, it has been estimated that 35–62% of athletes do not report every sustained concussion due to a combination of factors such as lack of knowledge, the pressure to play, and attitudes toward concussion [16]. Therefore, an anonymous retrospective survey may increase the likelihood of athletes reporting a concussion, particularly given that there is no associated perceived penalty for reporting. 

We also found that females were significantly more likely to experience a concussion while participating in BJJ when compared to males (*p* < 0.001). In the logistic regression model, gender remained significantly associated with increased odds of suffering a concussion while doing BJJ (Table 2). This is consistent with previous research, as studies have reported females to have a greater prevalence of concussions in multiple sports [9,11]. Our respondent pool consisted of 11.4% female BJJ practitioners (n = 89), which likely reflects the overall proportion in the sport (Table 1). In future studies, it will be important to follow a greater number of female BJJ practitioners to ensure the significance of observed trends. In contrast to concussions experienced as a result of BJJ participation, there was no significant difference between the reported concussion prevalence of males and females prior to participation in BJJ (X^2^ (2, n = 774) = 1.930, *p* = 0.381). This supports a lack of significant gender bias in reporting concussions within our study. If a gender bias did exist, we would expect to see an increase in reports of both concussions before and while engaging in BJJ. 

Incurring a concussion before BJJ was associated with increased odds of having a concussion during BJJ (Table 2). This is supported in the literature of other non-grappling sports; NCAA football players were shown to have a dose-dependent increased risk of concussion based on their previous self-reported concussion history [17]. It is also possible that practitioners who are more likely to report a concussion before BJJ are also more likely to report one when participating in BJJ. We attempted to control for this possibility with the anonymity of the survey. In a multiple variable logistic regression model, increased competition density was associated with increased odds for suffering a concussion during BJJ (Table 2). In a sport that has a wide spectrum of participants, from competitors to casual practitioners, it is likely that those who frequently compete for training also engage with greater intensity. This would be expected to lead to a greater incidence of head trauma. 

Among practitioners who had a concussion while engaging in BJJ, there were no increased odds for having a second concussion. We report 21.4% of concussions were recurrent, though this may underestimate the true value as we did not collect data on concussions beyond the second incident. This value is higher than the 8.2% recurrence reported by NCAA wrestling [11]. The difference may be secondary to sample size or to the enforcement of return to sports guidelines in the NCAA. 

The most common reported symptom of a concussion in this study was a headache, as reported by 75.7% of participants (Figure 1). Headache is typically the most commonly reported symptom by athletes who incur concussions [18,19,20]. Interestingly, the prevalence of concussion symptoms in BJJ was very similar to the results gathered by athletic trainers in high school and college football players. We report 27.6% and 9.9% of participants reporting memory problems and LOC, respectively (Figure 1). Among football players with concussions, 27.7% and 8.9% experienced amnesia and LOC, respectively [18]. Similar proportions of concussions with LOC have been seen in prospective studies in rugby [19]. This suggests that while concussion prevalence may change with data collection methodology, relative symptom prevalence is more consistent across collection methods and sports.

Clinically, the median time participants in this study reported resting after a concussion was three days. The median value was used rather than the mean due to outliers, including three practitioners who reported sitting out six months or greater. Current return to sports guidelines recommended by the Fifth International Conference on Concussion indicates that the amount of time sitting out should include a period of rest followed by the gradual return to sports where the participant remains below a symptom threshold [1]. Participants’ reported median return to sports time of three days, including 30.3% returning on the same day as being concussed. This suggests that there is a clear need to increase concussion education among BJJ coaches and practitioners (Figure 2). This is critical in a sport that does not require athletic trainers or medical professionals to monitor practice sessions. 

Given the methodology relied on retrospective surveying, there are limitations to this study that are typically seen with retrospective data. With the self-selection involved in survey participation, there is a possibility of systematic bias with over-reporting of concussions in the results. Conversely, by communicating with BJJ coaches and through social media, we may have overlooked individuals who no longer participate in BJJ due to their injury. Because participants are self-reporting injuries, there is a risk of recall bias and response bias. This is amplified by the attempt to gather lifetime concussion history from participants rather than a limited recall period. The fact that 52.2% of concussions before starting BJJ were reported to include a LOC raises concerns of recall bias. Several studies have shown that LOC does not take place in the majority of concussions [18,19,20]. It is likely that participants are underreporting the prevalence of concussions that did not include a LOC. The sample of 778 participants may not be representative of all BJJ practitioners. The survey did not ask BJJ practitioners to report the lifetime total number of concussions sustained. Future analysis should aim to include this value in order to determine the rate of concussions in BJJ. The survey distribution methodology resulted in the inability to estimate the number of participants the survey reached. Thus, we were unable to determine a response rate for the data collected. The study is also limited by the inability to assess the causes of concussions. We are unable to determine if concussions are more commonly associated with the impact onto a surface such as a mat or impact with an opponent. Likewise, we cannot determine if the rate of concussion is higher in practice or in competition. Many of these limitations would be addressed by performing a prospective analysis.

This study provides an initial investigation of concussions within BJJ. There is much research that needs to be done to better understand both concussions and practitioners within the sport. Future research could move in several directions, including a focus on female BJJ practitioners. Although it is difficult to make definitive conclusions without a larger sample size of female practitioners, this study suggests that females may be at an increased risk of concussions. In order to more accurately determine the risk of concussion in BJJ, a prospective injury surveillance study should be carried out using a representative cohort of BJJ practitioners. This will allow for tracking of athlete exposures in order to determine the rate of concussion while identifying risk factors and the mechanisms of concussions incurred while engaging in BJJ. BJJ includes takedowns, grappling while standing, and grappling on the ground. Determining which aspects of the sports may increase the risk of concussion could help determine the safest practices. 

## 5. Conclusions

This study provides an initial examination of sports-related concussion in BJJ practitioners. Through retrospective surveys, we have shown that 25.2% of BJJ practitioners reported having a concussion while engaging in BJJ. Female gender was associated with increased odds of incurring a concussion when compared to male counterparts. Practitioners who reported experiencing a concussion before beginning BJJ were associated with increased odds of incurring a concussion while engaging in BJJ. Among practitioners who reported having a concussion while engaging in BJJ, those who competed more times per year were associated with increased odds of incurring a second concussion. With a median return to sports time of three days and 30.3% of participants returning one the same day as incurring a concussion, there is a clear need for increased education among coaches and practitioners. Education on both concussion symptoms and return to sports guidelines are critical in BJJ where there typically are no athletic trainers or medical professionals monitoring the practice sessions.

## Figures and Tables

**Table 1:** Demographics of Respondents (n = 778).

Demographic Characteristics	Frequency (Percentage)
**Gender**	
Male	689 (88.6)
Female	89 (11.4)
**Age Group**	
18-23	154 (19.8)
24-28	163 (21.0)
29-34	153 (19.7)
35-41	157 (20.2)
42-49	106 (13.6)
50-67	46 (5.9)

**Table 2:** Logistic Regression Models for Experiencing a Concussion from Engaging in BJJ.

Variables in Equation	Odds Ratio	95% C.I.	*p*
Days per Week of Training	1.12	(0.94, 1.35)	0.211
Reported Concussion Before Starting BJJ	1.76	(1.14, 2.74)	0.011
Female Gender	1.95	(1.04, 3.65)	0.037
Lifetime Number of Competitions	1.03	(1.01, 1.04)	0.011
Competition Density	1.02	(1.00, 1.03)	0.021

## Supplementary Materials

The following are available online at https://www.mdpi.com/2075-4663/7/2/53/s1, Table S1: Brazilian Jiu-Jitsu Survey.
