# Supplementary material for: Epidemiology of Sports Related Concussion in Brazilian Jiu-Jitsu: A Cross-Sectional Study
Source: Sports (Basel). 2019 Feb 25;7(2):53. doi: 10.3390/sports7020053 (PMC6410117; doi:10.3390/sports7020053)
Supplement: Supplementary file 1 [file sports-07-00053-s001.zip › Survey.docx]

**Brazilian Jiu-Jitsu Survey**

Definition of Concussion: A concussion is a blow to the head or body followed by a variety of symptoms that may include any of the following: Headache, Dizziness, loss of balance, blurred vision, “seeing stars,” feeling in a fog or slowed down, memory problems, poor concentration, irritable, emotional outbursts, slurred speech, disturbed sleep pattern, nausea, or throwing-up. Getting “knocked-out” or being unconscious does NOT always occur with a concussion.

Email Address ________________________________

Age ________________________________

Gender ロ Male

ロ Female

ロ Other - What Gender? ______________

Did you have any concussions before ロ Yes

Starting Brazilian Jiu-Jitsu? ロ No

If so, How many concussions before _________________________________

Starting Brazilian Jiu-Jitsu (BJJ)?

How many times total before starting BJJ

Were you “knocked out” or unconscious _________________________________

From a concussion?

--------------------------------------------------------------------------------------------------------------------------------

--------------------------------------------------------------------------------------------------------------------------------

How long have you been training ____________________________

Brazilian Jiu-Jitsu (months/years)

On average, how many days per week ロ1 ロ2 ロ3

Have you trained BJJ (1-7)? ロ4 ロ5 ロ6

ロ7

How many BJJ competitions have you _____________________________

Participated in?

Did you have any concussions doing BJJ? ロ Yes

ロ No

(check “yes” if you think you might have had a concussion, even if you did not tell anyone)

Which Symptoms did you Experience? ロ Headache

ロ Dizziness

ロ Loss of Balance

ロ Blurred Vision

ロ Seeing Stars

ロ Feeling in a fog or slowed down

ロ Memory Problems

ロ Poor Concentration

ロ Nausea

ロ Throwing Up

ロ Lost Consciousness

What was your BJJ Belt Ranking at the ロ White

Time of your first concussion? ロ Blue

ロ Purple

ロ Brown

ロ Black

ロ Other

Other - what belt? ____________________

What was your age at the time of your

First concussion in BJJ? _____________________

Did you see a medical provider about your ロ Yes

Concussion? ロ No

ロ Not Applicable

After the Injury, how much time did you ______________________

Take before resuming BJJ training?

How long were you asymptomatic ロ 0 days ロ 1-3 days

(no headaches, dizziness, memory, ロ 4-6 days ロ 1-2 Weeks

or concentration problems) before ロ 2-4 weeks ロ > 1 month

Resuming BJJ? ロ Have Not Returned

Did you have another concussion after ロ Yes

Resuming training? ロ No

ロ Not Applicable

If you have any questions about the survey, please contact [BJJConcussions@gmail.com](mailto:BJJConcussions@gmail.com)
